# Identification and Investigation of miRNAs From *Gastrodia elata* Blume and Their Potential Function

**DOI:** 10.3389/fphar.2020.542405

**Published:** 2020-09-25

**Authors:** Chunxin Xia, Huaixiang Zhou, Xiaoyuan Xu, Tianlong Jiang, Shouliang Li, Dan Wang, Zuoming Nie, Qing Sheng

**Affiliations:** ^1^ College of Life Sciences and Medicine, Zhejiang Sci-Tech University, Hangzhou, China; ^2^ Zhejiang Provincial Key Laboratory of Silkworm Bioreactor and Biomedicine, Zhejiang Sci-Tech University, Hangzhou, China

**Keywords:** *Gastrodia elata* Blume, microRNA, high-throughput sequencing, expression profiles, A20, functional study

## Abstract

*Gastrodia elata* Blume (*G. elata*) is a valuable traditional Chinese medicine with neuroprotection, anti-inflammatory, and immune regulatory functions. MicroRNAs (miRNA) is a kind of endogenous noncoding small RNAs that plays distinctly important roles for gene regulation of organisms. So far, the research on *G. elata* is mainly focused on the pharmacological functions of the natural chemical ingredients, and the function of *G. elata* miRNA remains unknown. In this study, 5,718 known miRNAs and 38 novel miRNAs were identified by high-throughput sequencing from *G. elata*. Based on GO and KEGG analysis, we found that the human genes possibly regulated by *G. elata* miRNAs were related to the cell cycle, immune regulation, intercellular communication, etc. Furthermore, two novel miRNAs as Gas-miR01 and Gas-miR02 have stable and high expression in the medicinal tissues of *G. elata*. Further bioinformatics prediction showed that both Gas-miR01 and Gas-miR02 could target *Homo sapiens* *A20* gene, furthermore, the dual-luciferase reporter gene assay and Western Blotting verified the interaction of Gas-miR01 or Gas-miR02 with *A20*. These evidences suggested that *G. elata*-unique miRNAs might be involved in certain physiological processes. The animal experiment showed that Gas-miR01 and Gas-miR02 could be detected in some tissues of mice by intragastric administration; meanwhile, the A20 expression in some tissues of mice was downregulated. These results supported for the functional study of *G. elata* miRNAs.

## Introduction

*Gastrodia elata* (*G. elata*) Blume is a precious Chinese herbal medicine in China (Yuan et al., 2018). Many kinds of active ingredients in *G. elata* play an important role in the treatment of diseases. Since the 1950s, 81 compounds have been isolated from *G. elata*, including phenols, polysaccharides, and various sterols (Duan et al., 2013). Gastrodin is the main biologically active ingredient of *G. elata* (Liu et al., 2018).

In ancient China, *G. elata* was widely used to treat headache, dizziness, paralysis, epilepsy, joint pain, and numbness of limbs (Zhan et al., 2016). Some studies on its pharmacological properties have shown that *G. elata* has neuroprotective and antioxidant functions and could be used as an anti-inflammatory and antipsychotic drug (Kumar et al., 2013; Hu et al., 2014; Du et al., 2016; Wang et al., 2016). The pharmacological mechanisms of potential anticonvulsant activity of arrow *G. elata* have been extensively studied, which is similar to the mechanisms of known antiepileptic drugs (Wu et al., 2017). *G. elata* has also been found to treat anxiety, regulate the circulatory system, and improve memory (Niu et al., 2004). Additionally, *G. elata* could achieve its anti-aging effect by regulating certain signaling pathways (Song et al., 2016). Except for these, some chemical constituents such as anti-fungal protein GAFP- 1 in *G. elata* also play an important role. Certain phenols in *G. elata* can regulate the apoptosis-related signaling pathway by upregulating *Bcl-2* and protect the nerves of mice (Wu et al., 1996). Recent studies have shown that phenolic glycoside gastrodin, the main component of *G. elata*, showed potential neuroprotective effects by inhibiting neurotoxic proinflammatory mediators (Li et al., 2018). *G. elata* has a high potential medicinal value. However, research on *G. elata* in China and abroad mainly focuses on the pharmacological functions and product development of natural chemical constituents of *G. elata*. To date, there is no research report on the miRNA and its function in *G. elata*.

MicroRNAs (miRNAs) are conserved small RNA molecules, which can negatively regulate target genes at the transcriptional and posttranscriptional level through near-perfect complementarity to target mRNA (Nam et al., 2011; Razaviyan et al., 2018). Over the past decade, more and more reports have paid attention to their cross-kingdom regulation (Wang et al., 2014; Oghbaei et al., 2017; Zhou et al., 2017). The regulation of human target genes by rice MIR168a was first discovered in 2012 (Zhang et al., 2012). In 2013, Yang et al. demonstrated that plant-derived small RNAs are present in animal serum and urine through two independent studies (Yang et al., 2015a; Yang et al., 2015b). In 2014, Zhang et al. found that honeysuckle had little degradation of miR2911 after the decoction, and further experiments *in vitro* and *in vivo* showed that honeysuckle could target influenza A virus (IAV), thereby inhibiting its replication and reducing mouse mortality (Zhou et al., 2015). Furthermore, these studies demonstrate that storage, processing, and cooking do not eliminate plant miRNAs in food sources and that plant-derived miRNAs could survive in the simulated digestive system for at least 75 min (Philip et al., 2015). Additionally, miRNAs detected in human plasma were found to resist to ribonuclease A, suggesting that miRNA molecules may be accompanied by certain proteins, lipids, or other particles that protect them from degradation (Wang et al., 2012). To further explore the properties of small RNA through the placenta, Li *et al.* found that the level of small interfering RNA (siRNA) in mouse embryos increased significantly after feeding siRNA, and the level of alpha-fetoprotein (AFP) targeted by siRNA was downregulated (Wong et al., 2015). In addition, the factors like ultrasonic treatment, extreme heat, especially RNase treatment that could cause miRNA degradation, while being associated with plant molecules such as proteins and exosomes might be an effective way to protect miRNAs from degradation (Xie and Melzig, 2018). These studies confirmed the stability of plant-derived miRNAs during herb preparations, suggesting the possibility of medicinal plant miRNAs in mammals with complete function. The therapeutic potential for plant miRNAs has been confirmed by two different laboratories. A study reported that a mixture of three tumor suppressor miRNAs (simulated plant miRNAs) reduced colon tumor burden by oral administration in a mouse model of colon cancer (Mlotshwa et al., 2015). Another study found that plant miR159 was present in female serum, and its level was inversely correlated with breast cancer incidence and progression (Chin et al., 2016). Most of the identified miR159 are abundant in extracellular vesicles (EV), and *in vitro* experiments have shown that synthetic miR159 could reduce the proliferation of breast cancer cells (Chin et al., 2016). Furthermore, oral administration of the miR159 mimics significantly inhibited the growth of venous angiographic breast tumors in mice (Chin et al., 2016). Currently, many studies have provided evidence that exogenous miRNAs can play an important biological role in animals through oral administration.

In this study, using high-throughput sequencing, we comprehensively identified 5718 known miRNAs and 38 novel miRNAs from *G. elata* for the first time. The relative levels of these miRNAs in various stages of *G. elata* were further verified by qRT-PCR. The bioinformatics prediction showed these miRNAs could target the *Homo sapiens* genes, the dual-luciferase reporter gene assay and Western blotting indicated that both Gas-miR01 and Gas-miR02 could interact with the *A20* gene in 293T cell. As a zinc finger protein in the NF-κB signaling pathway, A20 is involved in cytokine-mediated immune and inflammatory responses, which has been proved to play a complex role impacting tumor development and therapeutic response according to some reports (Ma and Malynn, 2012; Santolla et al., 2018). Then, animal experiments also confirmed that Gas-miR01 and Gas-miR02 could be detected in some tissues of mice by intragastric administration with fresh *G. elata* total RNAs, *Gastrodia* decoction, and *Gastrodia* powder. Moreover, the A20 expression in some tissues of mice was downregulated in some tissues of mice. Our research provides a theoretical basis for the functional study of *G. elata*.

## Materials and Methods

### Sample Collection

**Figure 1:**
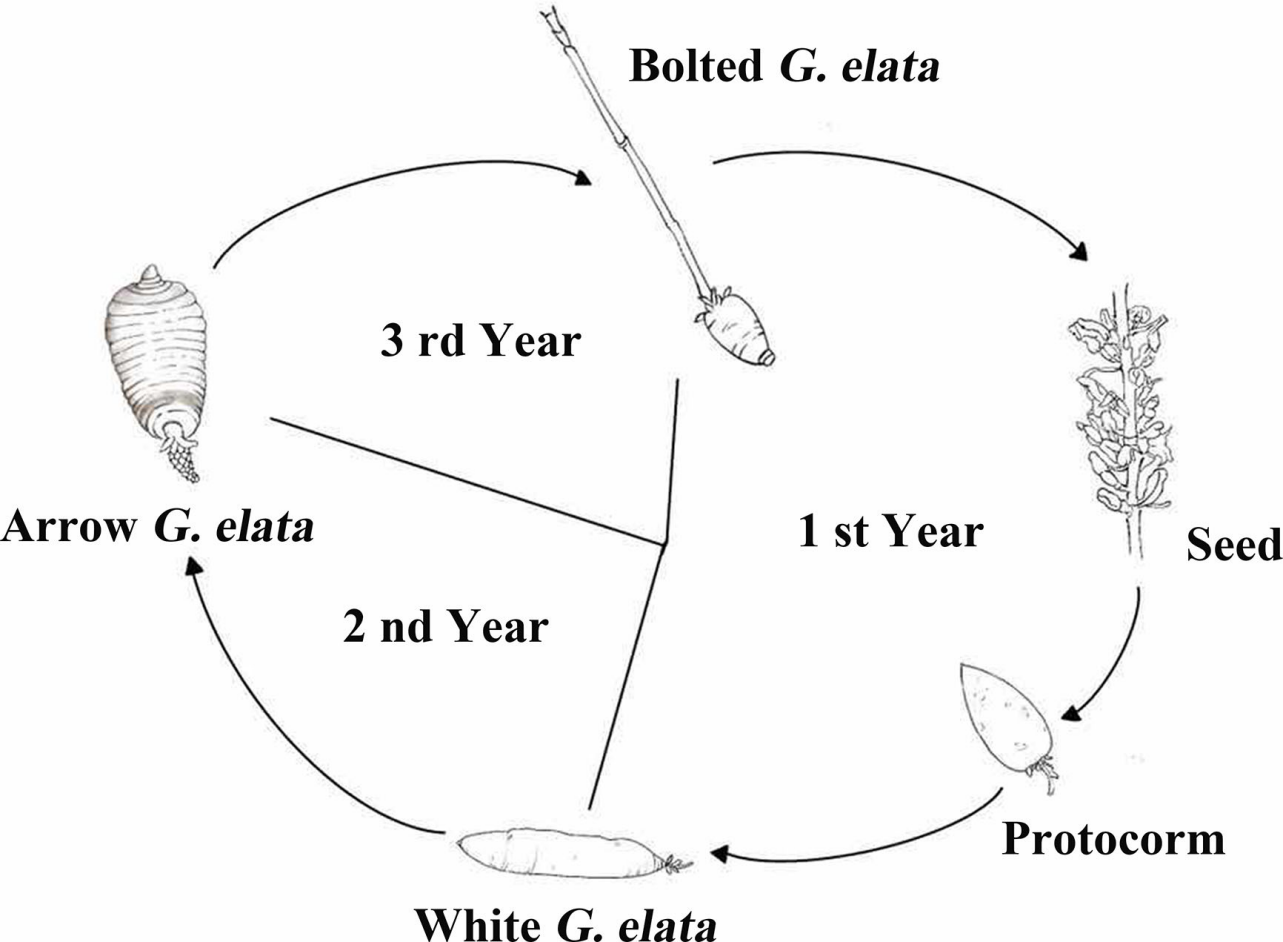
The life cycle of *G. elata* (trace diagram). The life cycle of *G. elata* has five stages, including *G. elata* seeds, protocorms, white *G. elata*, arrow *G. elata*, and bolted *G. elata*.

The life cycle of *G. elata* includes *G. elata* seeds, protocorms, white *G. elata*, arrow *G. elata*, and bolted *G. elata*, which is shown as **Figure 1**. The sample of fresh arrow *G. elata*, numbered as CL20151008, which was authenticated by Professor Zongsuo Liang in Zhejiang Provincial Key Laboratory of Plant Secondary Metabolism and Regulation, was collected at coordinate 115°93′12.5″E 31°27′50.0″N in Dabie Mountain, Anhui Province in China and stored at -80°C until total RNA was extracted. The voucher specimen is deposited at Herbarium of Northwest A&F University (WUK). After collecting the sample, *G. elata* seeds from fresh arrow *G. elata* were cultured. Protocorms, white *G. elata* and bolted *G. elata* were collected separately according to their growing stages.

In animal experiment, total RNAs of fresh *G. elata*, *Gastrodia* decoction and *Gastrodia* powder were used. *Gastrodia* superfine powder purchased from Yunnan Panlong Yunhai Ltd. Total RNA was extracted from fresh arrow *G. elata*. *Gastrodia* decoction was made by putting 150 mg of fresh arrow *G. elata* in 90 mL ddH_2_O, with cutting into pieces, soaking at room temperature for 30 min, simmering over low heat for 20 min to simulate traditional Chinese herbal decoction, finally getting 30 mL of *Gastrodia* decoction. *Gastrodia* powder was made as the follows: put 30 mg (the moisture content of arrow *G. elata* is 80%) of *Gastrodia* superfine powder in 30 mL ddH_2_O, and then blend to get a mixture called *Gastrodia* powder.

### RNA Extraction and qRT-PCR

Total RNAs extracted from different developmental stages from *G. elata* were reverse transcribed into the cDNAs using the Transcriptor First-Strand cDNA Synthesis Kit (Roche). The following program was used for the qRT-PCR using Fast Start Universal SYBR Green Master (Rox) (Roche): 95°C for 10 min, followed by 40 cycles of 95°C for 15 s and 60°C for 1 min, 95°C for 15 s, 60°C for 1 min, and 95°C for 15 s. Likewise, stem-loop qRT-PCR was used to detect miRNA (Specific RT-primer) using 5.8S rRNA as a normal control (Li et al., 2016). Likewise, total RNAs of mouse tissues in animal experiments were extracted. Then, the expression of Gas-miR01, Gas-miR02, and A20 were also detected by qRT-PCR using Fast Start Universal SYBR Green Master (Rox) (Roche). Primers for the quantitative real-time PCR and reverse transcription (**Supplementary materials Table 1**) were synthesized by Sangon Biotech (Shanghai). The data analysis was performed using ABI Prism 7500 SDS Software (Applied Biosystems, USA).

### Bioinformatics Analysis

We used the two-phase model (TPM) of *Gastrodia* to normalize the expression level. Normalized expression = (read count × 1,000,000)/libsize, where libsize refers to the number of reads on a miRNA compared to a sample (Zhou et al., 2010). We use DEGSeq for differential analysis (Wang et al., 2010) and count its Q-value (or adjust *p*-value) (Pounds and Cheng, 2004). The predicted target genes were subjected aligned against to GO and KEGG database, and the specific processes and pathways were emphatically analyzed (Nie et al., 2018). The GO enrichment analysis method refers to GOseq, and we counted the number of genes belonging to a certain GO number in the whole genome background and differentially expressed genes. Then, according to Fisher’s test, chi-squared test, and binominal test, the enrichment of GO entries in differentially expressed genes was determined relative to the background. RNAhybrid and miRanda softwares were used to predict differential miRNA target genes.

### Transfection With miRNA Mimics

Gas-miR01, Gas-miR02, and negative control were synthetized by Gene Pharma (Shanghai, China). 293T cells were cultured in six-well plates overnight in 37°C at 5% CO_2_, and Gas-miR01 and Gas-miR02 were overexpressed in 293T cells by transfection of the mimics for 20 pmol/1 × 10^5^ cells. The NC mimics was used as the negative control. Cells were collected 48 h post transfection for real-time qPCR analysis.

### Dual Luciferase Reporter Assay

The 293 T cells were cultured in a 24-well plate. And after 24 hours, the cells were transfected with pGL3-basic-A20 vector and co-transfected with Gas-miR01/Gas-miR02 mimics or negative control by Lipofectamine 3000TM transfection reagent (Thermo Scientific, MA, USA). The luciferase vectors were constructed by General Biol (Anhui, China). The sequence of Gas-miR01 and Gas-miR02 were as follows: Gas-miR01: 5’-GUUCAGGAAUGCUGUGGGAAG-3’; Gas-miR02: 5’-UUCAAUAAAGCUGUGGGAAA-3’. After 48 hours of transfection, the luciferase activity was detected by Dual-Luciferase Reporter Assay System (Promega, USA, E1910) according to the manufacturer’s protocol.

### Western Blotting

The 293T cells transfected with Gas-miR01/Gas-miR02/NC mimics were collected, and then the total proteins were extracted using the Cell Lysis Reagent (Roche). Total proteins were separated by 12% SDS-PAGE and transferred onto a polyvinylidene difluoride (PVDF) membrane (Millipore, USA) by electroblotting. After blocking with 5% skim milk in tris-buffered saline with Tween (TBST) (pH 7.4) for 2 h at room temperature, the protein was detected with an anti-A20 antibody (Cell Signaling Technology, USA) overnight at 4°C. The membrane was washed with TBST, and HRP-labeled (horseradish peroxidase-labeled) mouse anti-rabbit antibody was used as the secondary antibody (Cell Signaling Technology, USA). The signals were detected using the ECL Detection Kit (Advansta).

### Animal Experiments

Twenty-four female ICR mice were purchased from a pathogen-free animal facility at Zhejiang Chinese Medical University. The Institutional Review Board of Zhejiang Chinese Medical University approved all housing and surgical procedures. Animals were housed in cages (three to five mice per cage) and given free access to rodent chow and water ad libitum in a room maintained at 24°C and a 12-h/12-h light-dark cycle. At 7 weeks of age, each ICR mouse was fed with fresh *G. elata* total RNAs (30 μg/kg/2 h), *Gastrodia* decoction (3 mg/kg/2 h), *Gastrodia* powder (3 mg/kg/2 h), or double distilled H_2_O by gavage after fasting overnight. The mice were treated with total RNAs of fresh *G. elata* (30 μg/kg), *Gastrodia* decoction (3 mg/kg) and *Gastrodia* powder (3 mg/kg) by feeding a time per 2 h with six times during 12 h. After the sixth gavage for 0.5 h, tissues were collected, and total RNAs were extracted. The flow chart of animal experiments is shown in **Figure 6A**. All procedures used in this study were approved by the Zhejiang Sci-Tech University Animal Experimental Ethics Committee.

### Statistical Analysis

All data are the average of three separate experiments presented as the mean ± SEM. Data were analyzed by one-way ANOVA and LSD test with SPSS statistical analysis software.

## Results

### Length Distribution of *G. elata* Small RNA and Expression Profiles of miRNAs

**Figure 2:**
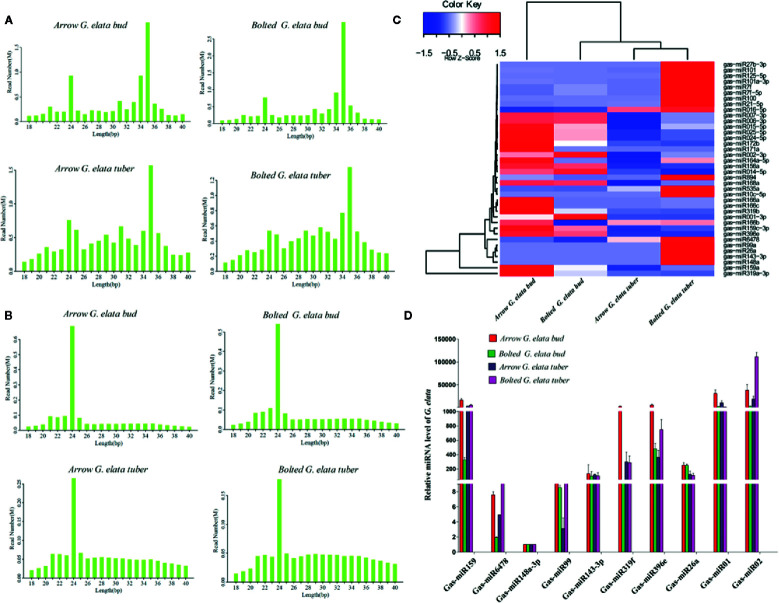
Length distribution of *G. elata* sRNA and expression profiles of 10 *G. elata* miRNAs in different tissues. Length distributions of clean total reads **(A)** and clean unique reads **(B)**. **(C)** Clustering map of difference miRNAs. **(D)** Relative expression levels of 10 *G. elata* miRNAs in different tissues.

In the clean total reads, the length distribution show higher peaks at 24 nt and 35 nt; in the clean unique reads, the highest peak of the length distribution is at 24 nt (**Figures 2A, B**). The length of miRNA and siRNA is approximately 24 nt, suggesting that the majority of *G. elata* small RNAs (sRNAs) were miRNAs or siRNAs, which may play an important role in regulating gene expression. Then, the expression of miRNAs from different *G. elata* tissues was explored. We firstly drew a hierarchical clustering map of 38 miRNAs with high expression and good reproducibility. The results are shown in **Figure 2C**. The arrow *G. elata* bud and bloted *G. elata* tuber showed a relative higher expression level of miRNAs. Eight known miRNAs (Gas-miR159, Gas-miR6478, Gas-miR148a-3p, Gas-miR99, Gas-miR143-3p, Gas-miR319f, Gas-miR396e, and Gas-miR26a) and two new miRNAs (Gas-miR01 and Gas-miR02) were selected to detect for expression levels (**Supplementary materials Table 2**). In traditional medicinal tissue of *G. elata* (arrow *G. elata* tuber), Gas-miR6478, Gas-miR148a-3p, Gas-miR99, Gas-miR143-3p, and Gas-miR26a had higher expression. Furthermore, the expression levels of 10 *G. elata* miRNAs were verified by qRT-PCR, and the qRT-PCR results were generally consistent with the sequencing results. It is worth mentioning that the two *G. elata*-unique miRNAs (Gas-miR01 and Gas-miR02) had relatively high expression levels in *G. elata* tissues (**Figure 2D**).

**Figure 3:**
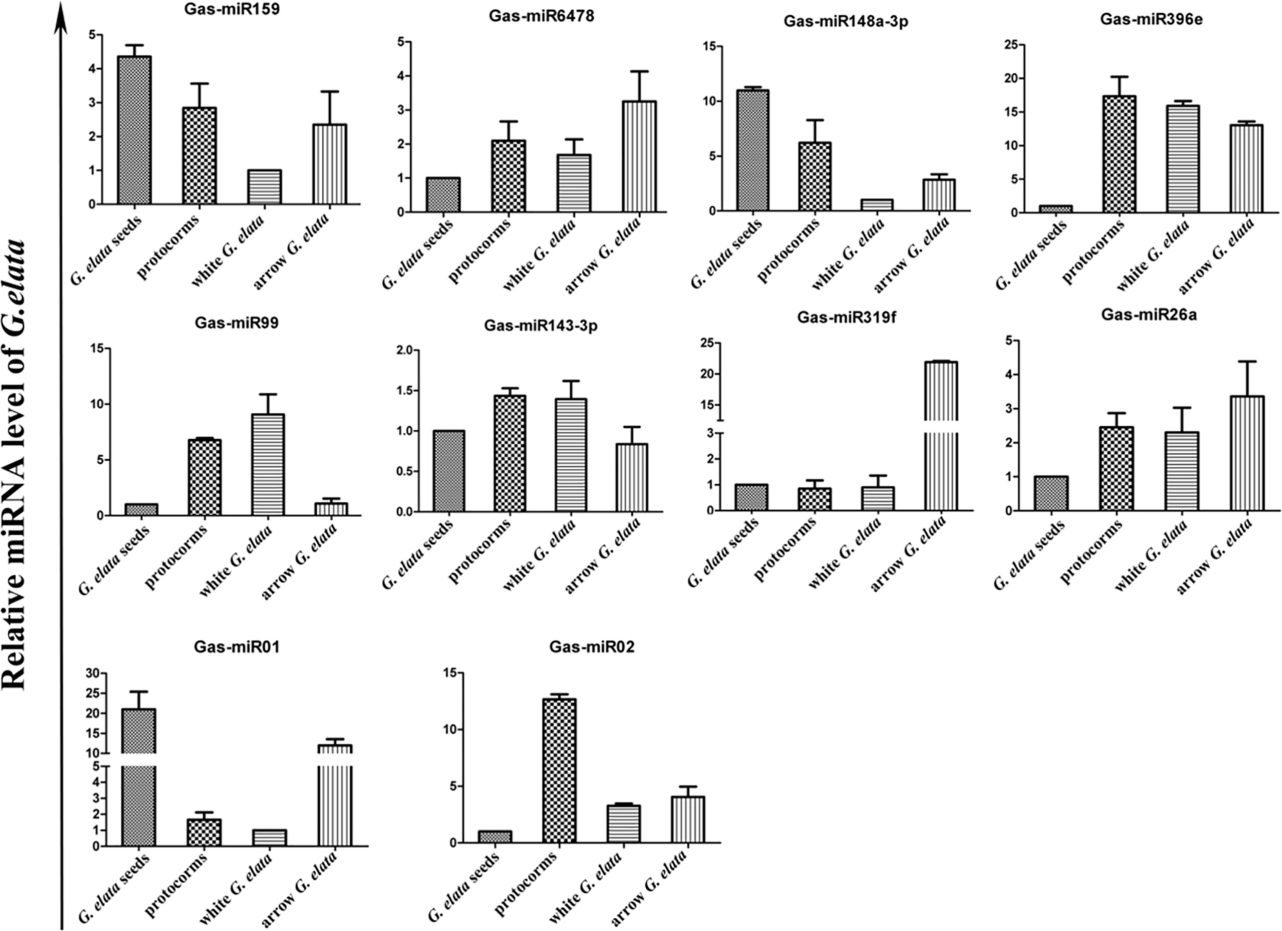
Expression profiles of 10 *G. elata* miRNAs in different growth stages. The relative *G. elata* miRNA expression levels in different growth stages were determined by qRT-PCR. The vertical coordinate means fold change.

The relative *G. elata* miRNA expression levels in different growth stages were also determined by qRT-PCR. The results showed that all the detected 10 miRNAs showed a different expression levels in different growth stages (**Figure 3**). On the whole, the Gas-miR01, Gas-miR319f and Gas-miR396e had relative high expression levels in certain growth stages. The *G. elata*-unique Gas-miR01 has lower expression levels in protocorms and white *G. elata* but higher expression levels in *G. elata* seeds and arrow *G. elata*. Compared with *G. elata* seeds, white *G. elata* and arrow *G. elata*, Gas-miR02 had the highest expression level in protocorms (**Figure 3**).

### GO and KEGG Enrichment Analysis of Differential miRNA Target Genes

**Figure 4:**
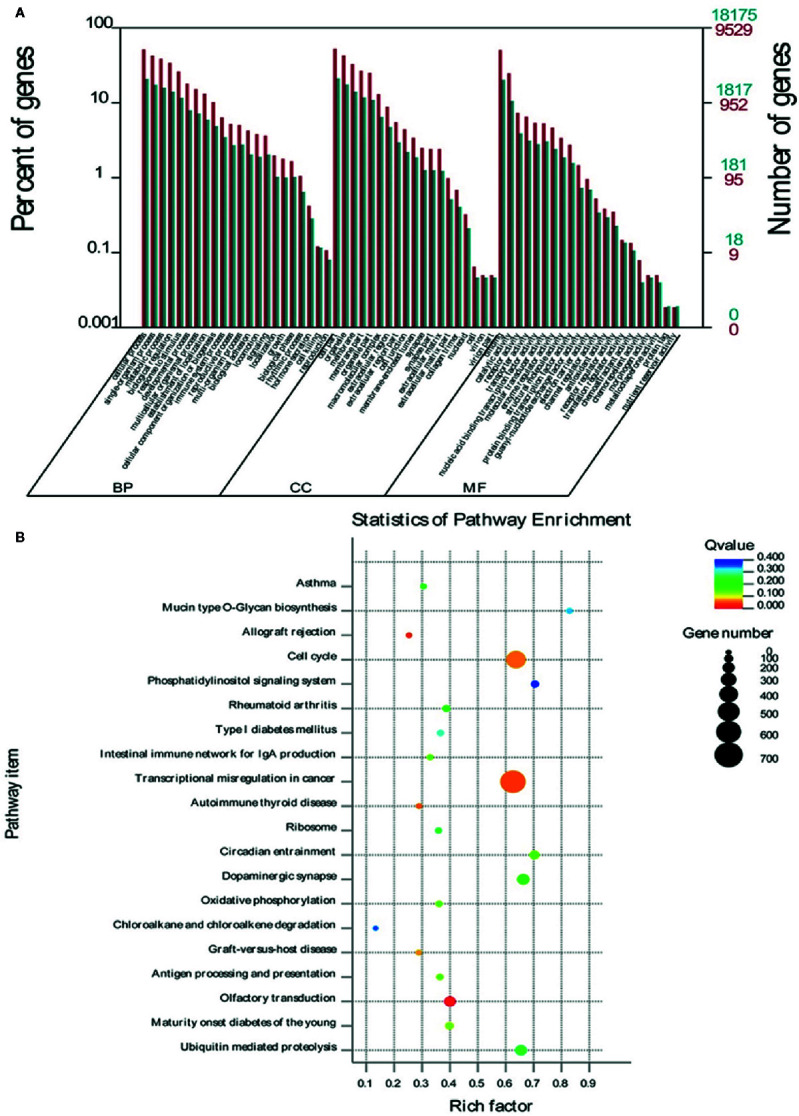
GO and KEGG enrichment analysis of differential miRNA target genes. **(A)** The target genes of miRNA involve BP (biological processes), CC (cellular components), and MF (molecular functions). **(B)** The closer the Q-value is to zero, the more significant the particular GO term associated with the group of genes; the closer the Q-value is to one, the opposite. The closer the Q-value is to 0, the more significant the particular GO term associated with the group of genes; the closer the Q-value is to one, the opposite.

To explore the potential functions of some specific miRNAs in *G. elata*, the potential *Homo sapiens* target genes of *G. elata* miRNAs were obtained by prediction using RNAhybrid and miRanda softwares. The candidate target genes were further analyzed by Gene Ontology analysis. In the medicinal tissue of *G. elata* (arrow *G. elata* tuber), the enrichment GO annotation of miRNA target genes involves biological regulation, immune system regulation, intercellular communication, and nucleic acid binding in three aspects: biological processes, cellular components, and molecular functions (**Figure 4A**). The KEGG pathway enrichment analysis indicated that the target genes annotation of the *G. elata* miRNAs is mainly enriched in the cell cycle, intestinal immune network, tumor transcriptional regulation, and olfactory signal transduction pathway (**Figure 4B**).

### The *G. elata*-Unique Gas-miR01 and Gas-miR02 Targeted the *Homo sapiens A20* Gene

**Figure 5:**
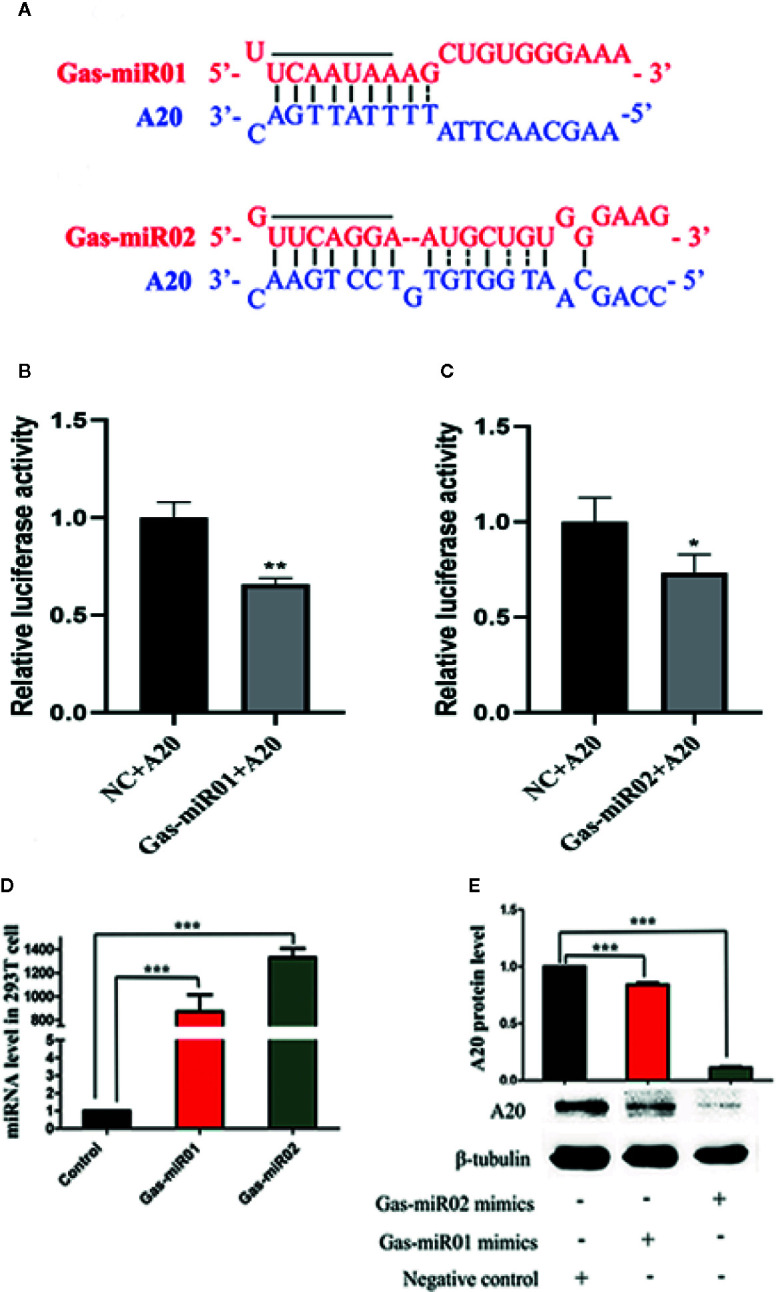
The down-regulation of A20 by Gas-miR01 and Gas-miR02. **(A)** Matching between Gas-miR01, Gas-miR02 and their target sites in *A20* gene. **(B)** The relative luciferase activity down-regulated by Gas-miR01. **(C)** The relative luciferase activity down-regulated by Gas-miR02. **(D)** Levels of Gas-miR01 and Gas-miR02 in 293T cells after transfected with corresponding mimics. **(E)** The expression level of A20 protein after transfected with Gas-miR01 and Gas-miR02 mimics. *p < 0.05; **p < 0.01; ***p < 0.001. Values are the mean ± SEM.

The bioinformatics analysis found that the seed sequences of the 5’ end of *G. elata-*unique miRNA Gas-miR01 and Gas-miR02 could perfectly match the targeted sites in the 3’ UTR region of the *Homo sapiens* *A20* gene (**Figure 5A**). Subsequently, the relative luciferase activity of pGL3-basic-A20 vector was significantly reduced when transfected with Gas-miR01 mimic or Gas-miR02 mimic, and this indicated that Gas-miR01 and Gas-miR02 could suppress the translation of A20 by interacting with the A20 gene (**Figures 5B, C**). The Gas-miR01 and Gas-miR02 mimics were then transfected into 293T cells successfully (**Figure 5D**), and the down-regulated expression level of A20 protein was detected by Western blotting (**Figure 5E**). The result showed that both Gas-miR01 and Gas-miR02 could significantly downregulate the expression of the protein A20 *in vitro*.

### Gas-miR01 and Gas-miR02 Were Detected in Some Tissues of Mice by Intragastric Administration

**Figure 6:**
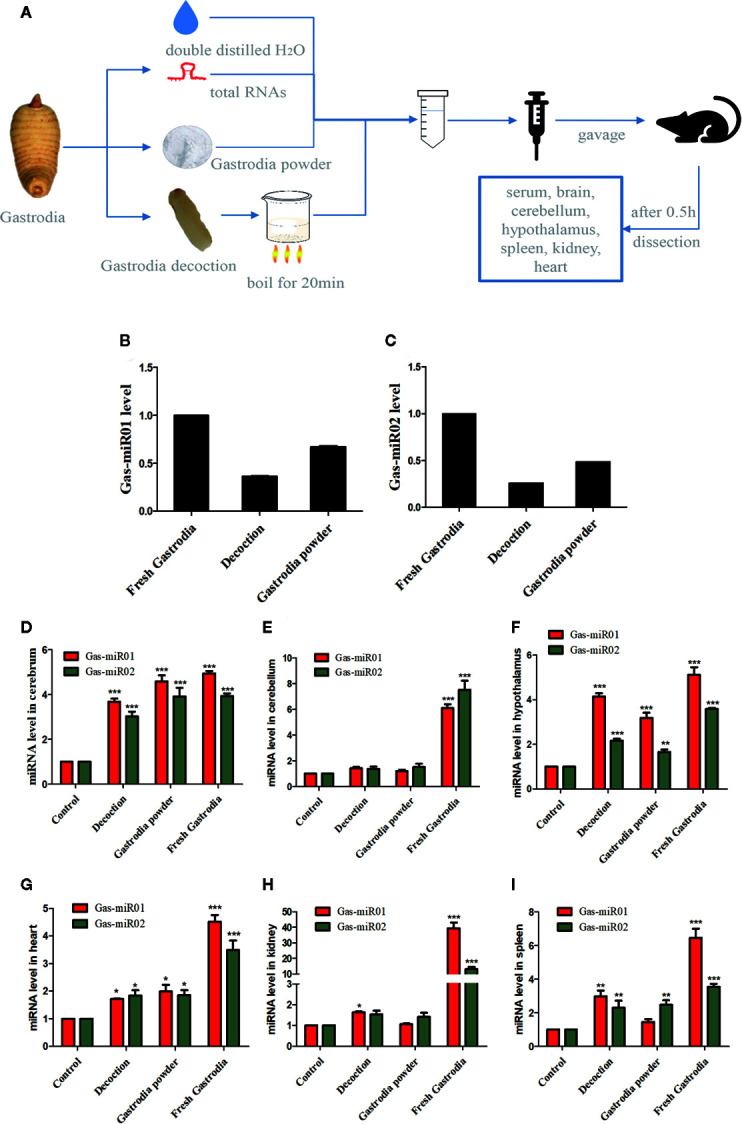
Gas-miR01 and Gas-miR02 were detected in some tissues of mice by intragastric administration. **(A)** Flow chart of animal experiments. The levels of Gas-miR01 **(B)** and Gas-miR02 **(C)** in fresh *G. elata* tuber, *Gastrodia* decoction and *Gastrodia* powder. The fresh *G. elata* total RNAs, *Gastrodia* decoction and *Gastrodia* powder were gavaged and the levels of Gas-miR01 and Gas-miR02 in various tissues of mice were detected by qRT-PCR. The levels of Gas-miR01 and Gas-miR02 in mouse tissues. **(D)** cerebrum; **(E)** cerebellum; **(F)** hypothalamus; **(G)** heart; **(H)** kidney; **(I)** spleen. *p < 0.05; **p < 0.01; ***p < 0.001 compared with the control group, n = 6. Values are the mean ± SEM.

**Figure 7:**
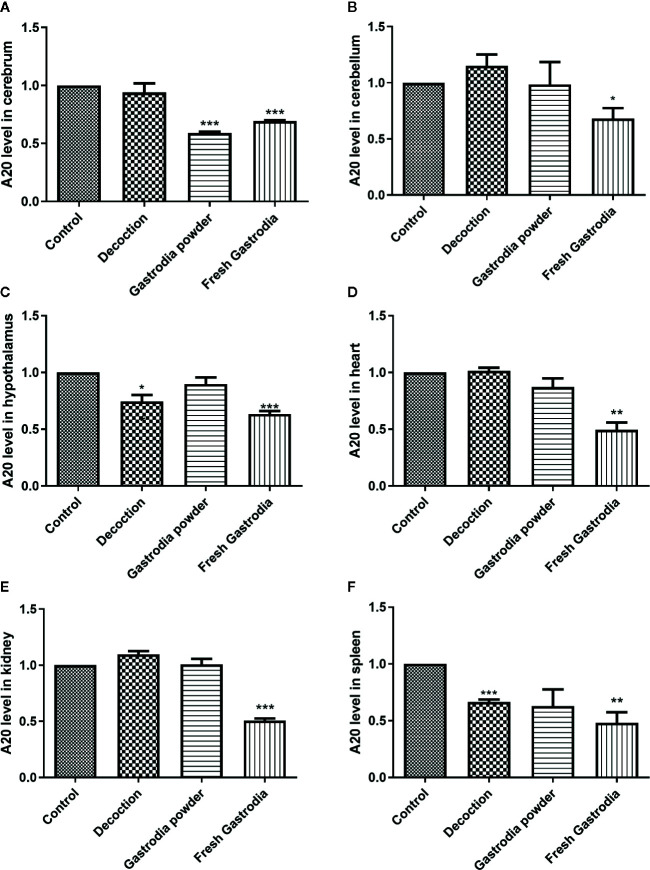
The A20 expression was downregulated in many tissues of mouse. The result of qRT-PCR showed the mRNA level of *A20* in various tissues of mice. **(A)** Cerebrum. **(B)** Cerebellum. **(C)** Hypothalamus. **(D)** Heart. **(E)** Kidney. **(F)** Spleen. *p < 0.05; **p < 0.01; ***p < 0.001 compared with the control group, n = 6. Values are the mean ± SEM.

The total RNAs were extracted from fresh *G. elata* tuber, *Gastrodia* decoction, and *Gastrodia* powder, respectively, and both Gas-miR01 and Gas-miR02 were all detected in them by using qPCR (**Figures 6B, C**). To further determine the cross-kingdom regulation of *G. elata* miRNAs, the fresh *G. elata* total RNAs, *Gastrodia* decoction *Gastrodia* powder, and double distilled H_2_O were gavaged. The levels of Gas-miR01 and Gas-miR02 in various tissues of mice were detected by qRT-PCR (**Figures 6D–I**). Compared with the control, the levels of Gas-miR01 and Gas-miR02 in the brain, cerebellum, hypothalamus, heart, kidney, and spleen of mice were significantly increased after total RNAs were gavaged, especially in the kidneys. After the *Gastrodia* powder and *Gastrodia* decoction were gavaged, the levels of Gas-miR01 and Gas-miR02 in the brain, cerebellum, and spleen tissues of mice also increased significantly. These results indicated that Gas-miR01 and Gas-miR02 could be into the mouse internal environment through the gastrointestinal tract and could be detected in some tissues of mice. Furthermore, qRT-PCR showed the A20 expression was also downregulated in some tissues of mice (**Figures 7A–F**).

## Discussion

miRNA, a small endogenous RNA, plays an important role by targeting specific mRNAs in plants, animals and humans (Sala-Cirtog et al., 2015). Studies have shown that many plant miRNAs are indispensable in cell proliferation and the secondary process of plant secondary metabolism. miRNA detection analysis displayed that Gas-miR319f had slightly different expression levels in *G. elata* seeds, protocorms, and white *G. elata*, but it would rise sharply 20 times to the original in arrow *G. elata*, which is the main medicinal tissue of *G. elata*. Therefore, we inferred that the sharp increase in the level of Gas-miR319f during this stage might be related to the large amount of synthetic medicinal components, such as gastrodin.

In China, the traditional medicinal herbs have been used for thousands of years and are usually decocted and orally administrated. A growing body of research suggests that the molecular mechanisms of medicinal herbs have been revealed. Since the discovery of plant miRNAs in human tissues and serum after ingestion, the relationship between the two kingdoms has been presented in a new perspective (Han and Luan, 2015). For example, miRNA therapy might be the next best step in determining new treatment options for medicinal plants (Thakur et al., 2014; Bai et al., 2018). Plant miRNAs could be detected in the sera and tissues of various animals, and these exogenous miRNAs were primarily acquired orally through food intake (Zhang et al., 2012). In addition to the findings on transfer of miRNAs and their therapeutic applications, similar studies could provide evidences for further research that might influence modern definitions of herbs. A recent study showed that synthetic MIR2911, extracted RNA from honeysuckle decoction, and honeysuckle decoction all significantly inhibited H1N1 viral replication and rescued viral infection-induced mouse weight loss. The results indicated that MIR2911 is an active component identified in traditional Chinese medicine to directly target various IAVs and may represent a novel type of natural product that effectively suppresses viral infection (Zhou et al., 2015).

In our research, the results suggested that the human genes related to immune system regulation could be regulated by *G. elata* miRNAs, and *in vitro* experiment evidenced that both Gas-miR01 and Gas-miR02 mimics could downregulate the expression of the *Homo sapiens* A20, the key protein in the NF-κB signaling pathway. NF-κB is present in many eukaryotic cells, widely involved in many physiological processes in humans, and related to many diseases such as Parkinson’s disease, Alzheimer’s disease and fatty liver disease (Lee and Ambros, 2001; Gommans and Berezikov, 2012; Ge et al., 2016; Bazazzadegan et al., 2017). And according to some reports, A20 expression is increased in a number of solid human tumors and cancers, which likely contributes to both carcinogenesis and response to chemotherapy. These evidences uncovered the complexities of the mechanisms involved in A20’s impact on tumor development and response to treatment (Hjelmeland et al., 2010; Bellail et al., 2012; Dong et al., 2012; Qiao et al., 2015). Animal experiments showed that the *G. elata*-unique miRNAs as Gas-miR01 and Gas-miR02 could be detected in both *Gastrodia* decoction and *Gastrodia* powder, suggesting these two *G. elata-*unique miRNAs could still remain stable after decoction and long-time storage. Furthermore, Gas-miR01 and Gas-miR02 could be detected in some tissues of mice by intragastric administration of the total RNAs of fresh *G. elata* tuber, *Gastrodia* decoction, and *Gastrodia* powder; meanwhile, the A20 expression in some tissues of mice was also downregulated. These evidences suggest that *G. elata* miRNAs could be into the mouse internal environment and conductcross-kingdom regulation. The above results provided a new theoretical basis for the further study on the cross-kingdom regulation of *G. elata* miRNAs. Our findings may provide data support for the functional study of miRNAs and the medicinal ingredients in *G. elata* in the future.

## Conclusion

In this study, we firstly obtained 5,718 known miRNAs and 38 *G. elata*–specific miRNAs by high-throughput sequencing. The expression levels of the miRNAs in various stages and tissues of *G. elata* were different by qRT-PCR, which was found to be high expression in the medicinal tissue of *G. elata* (arrow *G. elata* tuber). Bioinfomatics analysis indicated that *G. elata* miRNAs might be involved in the cross-kingdom regulation of *Homo sapiens* genes. It was confirmed for the first time that the two *G. elata*-unique miRNAs Gas-miR01 and Gas-miR02 could downregulate the expression of the *Homo sapiens* A20 gene *in vitro*. Furthermore, *G. elata*-unique miRNAs Gas-miR01 and Gas-miR02 could be detected in some tissues of mice through the gastrointestinal tract, and A20 expression was downregulated. These findings lay a foundation for the study of the cross-kingdom regulation of *G. elata* miRNAs.

## Data Availability Statement 

All datasets generated for this study are included in the article/supplementary material.

## Ethics Statement

The animal study was reviewed and approved by Zhejiang Sci-Tech University Animal Experimental Ethics Committee.

## Author Contributions

QS designed the project and instructed all the process. CX and HZ designed and performed the main experiments and wrote the article under the guidance of QS. XX and TJ undertook part of the experiments and analyzed the data. SL participated in the research and helped to write the article. DW and ZN helped to design the project and revise articles.

## Funding

This work was supported by grants from Project of Public Welfare Technology Research in Zhejiang Province (grant numbers LGF18H250004 and LGF19H250002).

## Conflict of Interest

The authors declare that the research was conducted in the absence of any commercial or financial relationships that could be construed as a potential conflict of interest
