# Supplementary material for: Identification and Investigation of miRNAs From Gastrodia elata Blume and Their Potential Function
Source: Front Pharmacol. 2020 Sep 25;11:542405. doi: 10.3389/fphar.2020.542405 (PMC7545038; doi:10.3389/fphar.2020.542405)
Supplement: Supplementary file 1 [file DataSheet_1.docx]

Supplementary Material

Supplementary Table 1 Primers were used for cDNA synthesis and the real-time quantitative PCR experiment

| Gene | RT-qPCR forward primer(5’-3’) | | RT-qPCR reversed primer(5’-3’) |
| --- | --- | --- | --- |
| Gas-miR159 | CGGGTTTGGATTGAAGGG | | CAGTGCAGGGTCCGAGGTAT |
| Gas-miR6478 | GCGCCGACCTTAGCTCAGT | | CAGTGCAGGGTCCGAGGTAT |
| Gas-miR148a-3p | CGGGCTCAGTGCACTACAGAA | | CGCAGGGTCCGAGGTATT |
| Gas-miR99 | TGCCAACCCGTAGATCCG | | CGCAGGGTCCGAGGTATT |
| Gas-miR143-3p | AGCGGTGAGATGAAGCACTGT | | CGCAGGGTCCGAGGTATT |
| Gas-miR319f | CGGCGTTGGACTGAAGGG | | CGCAGGGTCCGAGGTATT |
| Gas-miR396e | CGGGTTCCACAGCTTTCTTG | | CGCAGGGTCCGAGGTATT |
| Gas-miR26a | CGGGCTTCAAGTAATCCAGG | | CAGTGCAGGGTCCGAGGTAT |
| Gas-miR01 | TCGGGTTCAGGAATGCTGTG | | CAGTGCAGGGTCCGAGGTAT |
| Gas-miR02 | GCGGGTTCAATAAAGCTGTG | | CAGTGCAGGGTCCGAGGTAT |
| *A20* | TGGGAGCAGTGTTAAAGGCA | | TCGGAACTGTGGGCAAAACT |
| *GADPH* | AGTGTGACGTTGACATCCGT | | GCAGCTCAGTAACAGTCCGC |
| *5.8SrRNA* | AAACGACTCTCGGCAACGG | | GCGCAACTTGCGTTCAAAG |
| Gene | | stem-loop primers | |
| Gas-miR159 | | GTCGTATCCAGTGCAGGGTCCGAGGTATTCGCACTGGATACGACCAGAGC | |
| Gas-miR6478 | | GTCGTATCCAGTGCAGGGTCCGAGGTATTCGCACTGGATACGACCACCAA | |
| Gas-miR148a-3p | | GTCGTATCCAGTGCAGGGTCCGAGGTATTCGCACTGGATACGACCAAAGT | |
| Gas-miR99 | | GTCGTATCCAGTGCAGGGTCCGAGGTATTCGCACTGGATACGACCACAAG | |
| Gas-miR143-3p | | GTCGTATCCAGTGCAGGGTCCGAGGTATTCGCACTGGATACGACGAGCTA | |
| Gas-miR319f | | GTCGTATCCAGTGCAGGGTCCGAGGTATTCGCACTGGATACGACAGGGAG | |
| Gas-miR396e | | GTCGTATCCAGTGCAGGGTCCGAGGTATTCGCACTGGATACGACACAGTT | |
| Gas-miR26a | | GTCGTATCCAGTGCAGGGTCCGAGGTATTCGCACTGGATACGACCACAGC | |
| Gas-miR01 | | GTCGTATCCAGTGCAGGGTCCGAGGTATTCGCACTGGATACGACCTTCCC | |
| Gas-miR02 | | GTCGTATCCAGTGCAGGGTCCGAGGTATTCGCACTGGATACGACTTTCCC | |

Supplementary Table 2 10 miRNA sequences to be verified

| Gene | Sequence(5’-3’) |
| --- | --- |
| Gas-miR159 | UUUGGAUUGAAGGGAGCUCUG |
| Gas-miR6478 | CCGACCUUAGCUCAGUUGGUG |
| Gas-miR148a-3p | UCAGUGCACUACAGAACUUUG |
| Gas-miR99 | AACCCGUAGAUCCGAUCUUGUG |
| Gas-miR143-3p | UGAGAUGAAGCACUGUAGCUC |
| Gas-miR319f | UUGGACUGAAGGGAGCUCCCU |
| Gas-miR396e | UUCCACAGCUUUCUUGAACUGU |
| Gas-miR26a | UUCAAGUAAUCCAGGAUAGGCUGUG |
| Gas-miR01 | GUUCAGGAAUGCUGUGGGAAG |
| Gas-miR02 | UUCAAUAAAGCUGUGGGAAA |
