# Supplementary material for: Identification and Investigation of miRNAs From Gastrodia elata Blume and Their Potential Function
Source: Front Pharmacol. 2020 Sep 25;11:542405. doi: 10.3389/fphar.2020.542405 (PMC7545038; doi:10.3389/fphar.2020.542405)
Supplement: Supplementary file 2 [file DataSheet_2.docx]

The Botanical Scans

The sample of fresh *Gastrodia elata Blume*, numbered as CL20151008, which was authenticated by Professor Zongsuo Liang (full details are given in Appendix), was collected at coordinate 115°93′12.5″E 31°27′50.0″N in Dabie Mountain, Anhui Province in China, stored at -80°C in Zhejiang Provincial key laboratory of plant secondary metabolism and regulation.


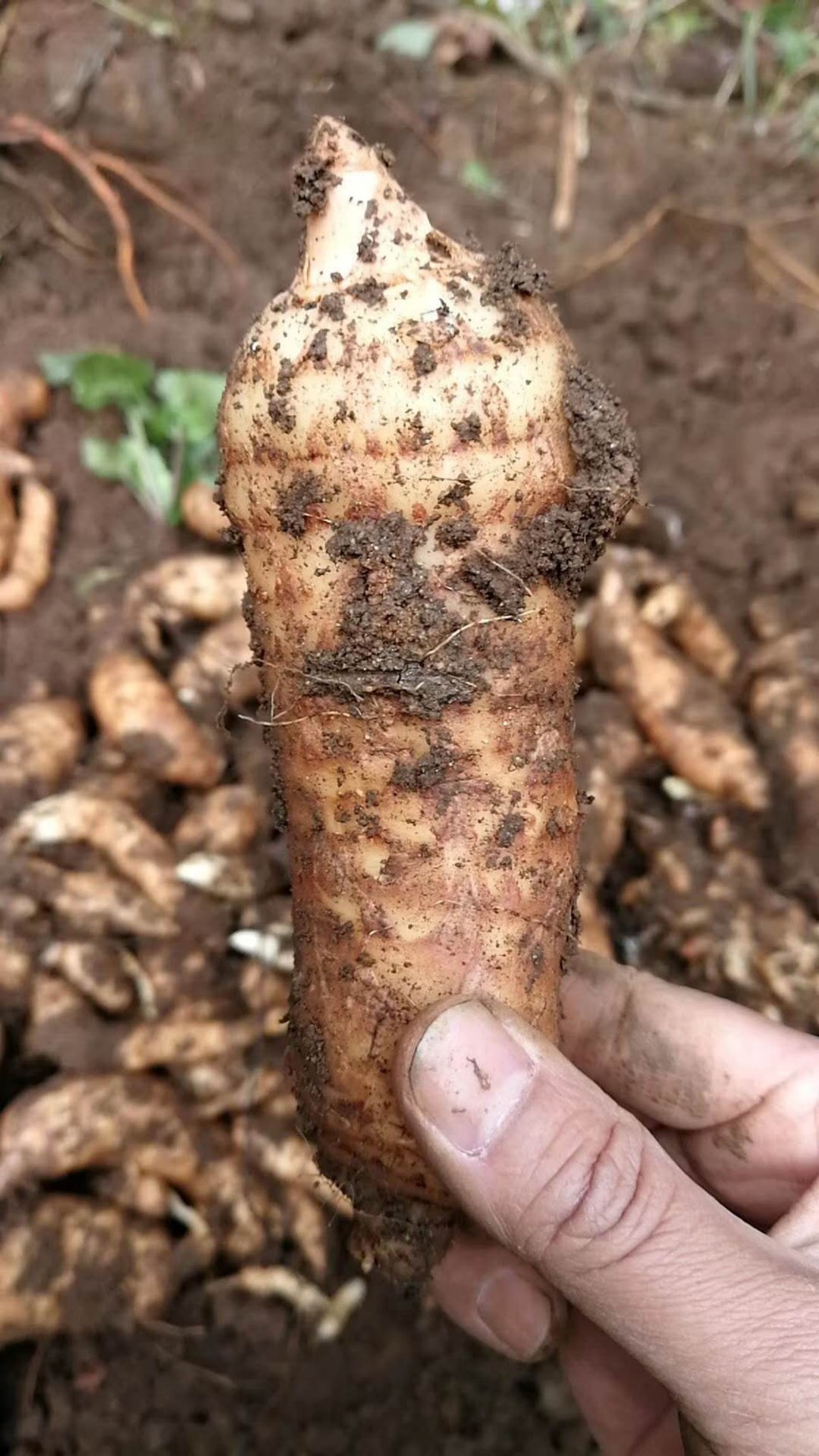


Fig.1 Tuber 1 of *Gastrodia elata Blume*


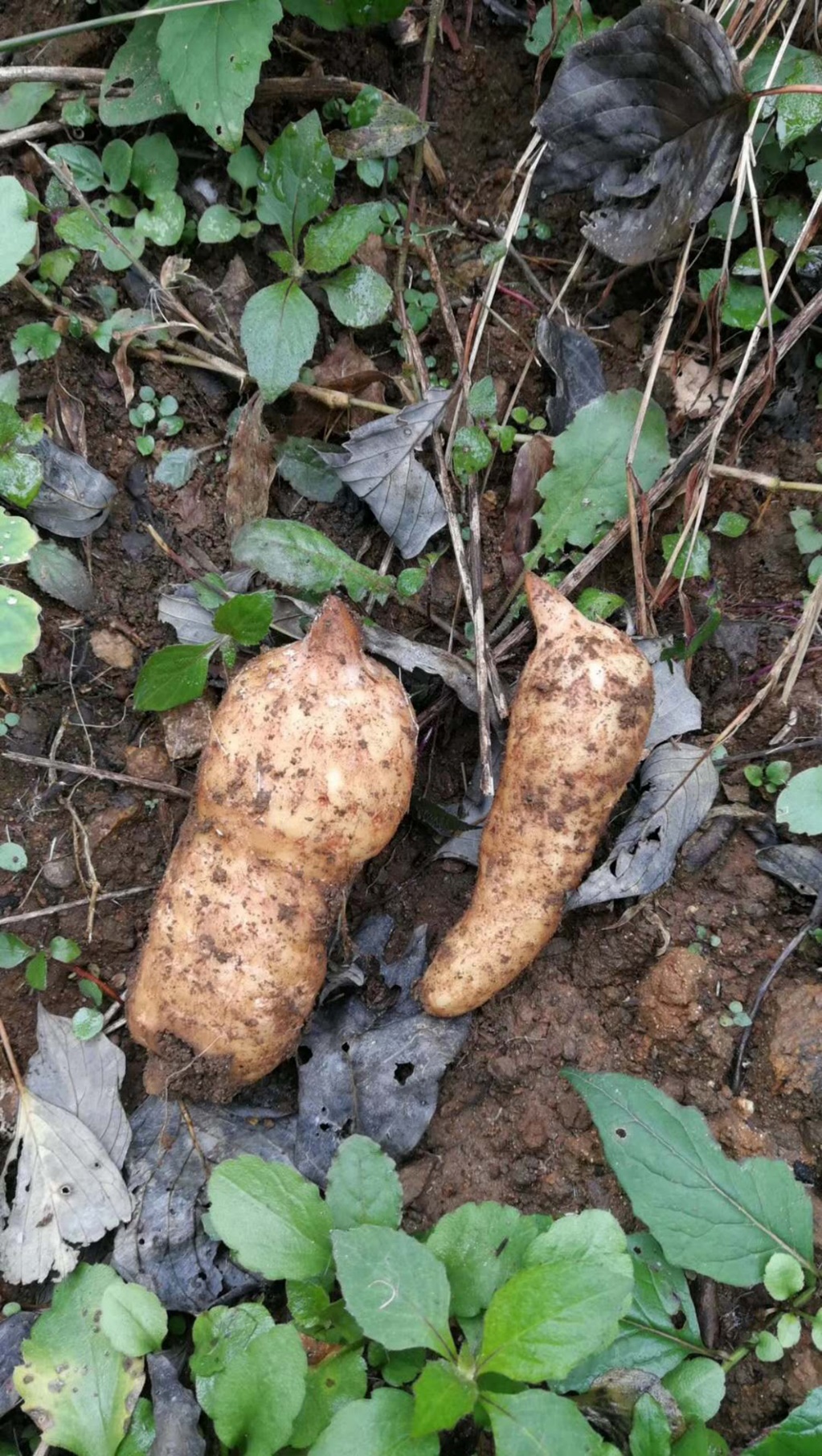


Fig.2 Tuber 2 of *Gastrodia elata Blume*


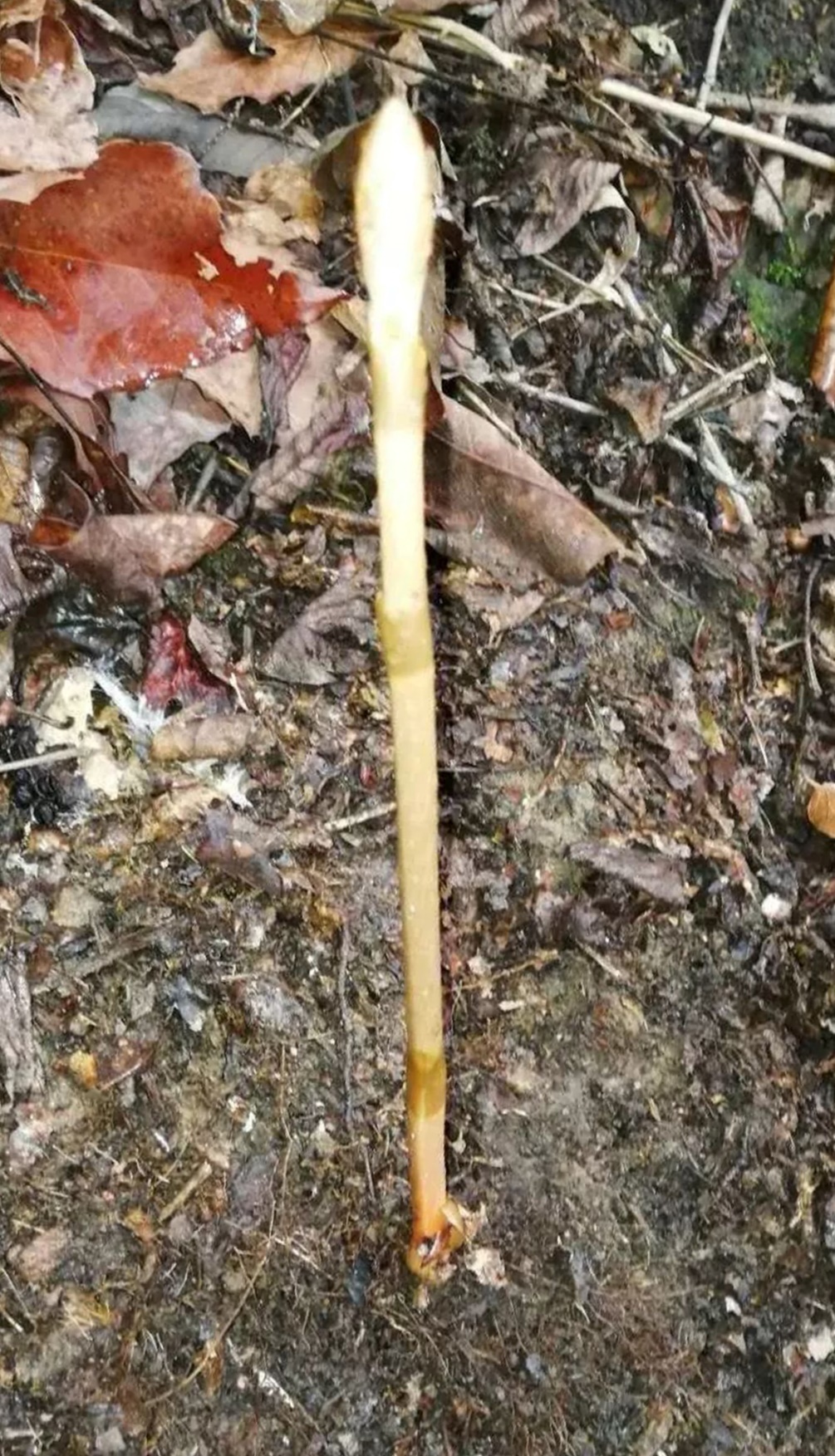


Fig.3 The stem of *Gastrodia elata Blume*
